# Bactericidal metabolites from *Phellinus noxius* HN-1 against *Microcystis aeruginosa*

**DOI:** 10.1038/s41598-017-03440-2

**Published:** 2017-06-09

**Authors:** Pengfei Jin, Haonan Wang, Wenbo Liu, Shujian Zhang, Chunhua Lin, Fucong Zheng, Weiguo Miao

**Affiliations:** 1 College of Environment and Plant Protection, Hainan University, Haikou, 570228 China; 2 Department of Plant Pathology, University of Florida, Gainesville, FL 32611-0680 United States of America

## Abstract

Harmful algal blooms cause serious problems worldwide due to large quantities of cyanotoxins produced by cyanobacteria in eutrophic water. In this study, a new compound named 2-(3, 4-dihydroxy-2-methoxyphenyl)-1, 3-benzodioxole-5-carbaldehyde (Compound **1**), together with one known compound, 3, 4-dihydroxybenzalacetone (DBL), was purified from *Phellinus noxius* HN-1 (CCTCC M 2016242). Compound **1** and DBL displayed activity against the cyanobacteria *Microcystis aeruginosa* with a half maximal effective concentration of 21 and 5 μg/mL, respectively. Scanning electron and transmission electron microscopic observations showed that the compounds caused serious damage and significant lysis to *M. aeruginosa* cells. qRT-PCR assay indicated that compound **1** and DBL exposure up-regulated the expression of gene *mcyB* and down-regulated the expression of genes *ftsZ*, *psbA1*, and *glmS* in *M. aeruginosa*. This study provides the first evidence of bactericidal activity of a new compound and DBL. In summary, our results suggest that compound **1** and DBL might be developed as naturally-based biocontrol agents.

## Introduction

Harmful algal blooms (HABs) are considerable problems worldwide because of large quantities of toxins produced by cyanobacteria in eutrophic water^1–4^. It causes economic losses to aquaculture industry, water pollution, and harms to wildlife and human health^5, 6^. However, traditional control strategies usually induce the secondary pollution due to the release of phytotoxins^6–8^.

A growing body of evidence suggests that biological products can control harmful algae such as *Microcystis aeruginosa* ^9^. Therefore, finding new bioactive compounds by screening microbial secondary metabolites has become increasingly important. Algicidal bacteria, such as *Alteromonas, Bacillus, Cytophaga, Micrococcus, Pseudomonas*, and *Vibrio*, are algal species-specific in most cases and their abundance has been found to increase during the decline of an algal bloom^7, 10, 11^. Those bacteria are inhibitory to harmful algae and affect their algal properties, such as toxin production^12, 13^. Previous studies have shown that those bacteria may produce extracellular algicidal substances, such as the β-cyano-L-alanine produced by *Vibrio* spp., 1-methyl-β-carboline and phenazine pigments from *Pseudomonas* spp., and lactones from *Ruegeria pomeroyi* ^14–16^. In addition, some plants chemicals such as ellagic acid, eugeniin^17^, ethyl-2-methylacetoacetate^18^, gallic acids, nonanoic acid, and pyrogallol, (+)-catechin^19^ that have anti-cyanobacteria activities. Two compounds isolated from the endophytic fungi *Seimatosporium* sp. and *Microsphaeropsis* sp. have powerful algicidal properties^20^. To the best of our knowledge, there have been few similar reports on algicidal substances isolated from fungi. Moreover, the inhibition mechanisms remain elusive even though some substances have been reported to control *M*. *aeruginosa*.

In the previous study, *Phellinus noxius* HN-1 stored in our lab^21^ was tested a variety of biological activities and could inhibit the growth of *M. aeruginosa* (unpublished). In this study, we purified a new compound and 3, 4-dihydroxybenzalacetone (DBL) from *P. noxius* HN-1. We evaluated the potential bactericidal properties of these two compounds, and the effects on cell morphology of *M. aeruginosa*. In order to dissect the inhibition mechanisms of the compounds, we conducted genes expression survey using the microcystin peptide synthesis gene *mcyB*, cell division gene *ftsZ*, photosynthesis gene *psbA1*, and peptidoglycan synthesis gene *glmS*.

## Results

### Purification of compounds

**Figure 1:**
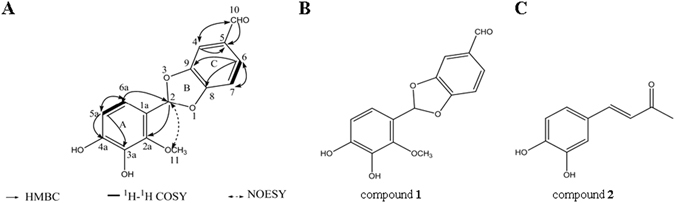
The structures of compound **1** and DBL. (**A**) Key HMBC, ^1^H-^1^H COSY, and NOESY correlations of compound **1**, (**B**) The structure of compound **1**, (**C**) The structure of DBL.

Compound **1** was isolated as a brown crystal. Its molecular formula was assigned as C_15_H_12_O_6_ from its high resolution-electron spin ionization-mass spectrometry (HR-ESI-MS) with a molecular ion at m/z 289.2638 [M + H]^+^ (see Supplementary Fig. S1), UV (MeOH) λmax (log ε) 207. The IR spectrum displayed the presence of, phenyl (1580 cm^−1^), methyl (2953 cm^−1^), methylene (2923 cm^−1^), aldehyde (1719 cm^−1^). The NMR data (Table 1) indicated ten degrees of unsaturation. The ^1^H NMR spectrum of compound **1** (Table 1 and see Supplementary Fig. S2) showed the presence of one oxygenated methine proton [δ 5.18 (1 H, s, H-2)], five aromatic protons [δ 7.28 (1 H, d, *J* = 1.6 Hz, H-4), δ 7.27 (1 H, d, *J* = 1.6 Hz, 6.6 Hz, H-6), δ 6.89 (1 H, d, *J* = 8.6 Hz, H-7), δ 6.82 (1 H, d, *J* = 8.2 Hz, H-5a), δ 6.71 (1 H, d, *J* = 9.8 Hz H-6a)], one aldehyde group [δ 9.65 (1 H, s, H-10)], and one methoxy group [δ 3.32 (3 H, s, H-11)]. The ^13^C NMR spectrum of compound **1** (see Supplementary Fig. S3) showed 15 carbon signals, including a methoxy group (CH_3_O-11, δ_C_ 49.8), one aldehyde group (CHO-10, δ_C_ 193.1), one methine group (C-2, δ_C_ 104.8), and 12 olefinic carbons (δ_C_ 115.8, 131.1, 126.4, 116.2, 147.1, 153.7, 146.6, 115.3, 146.1, 130.8, 114.8, and 119.4) ascribed for two phenyl groups. Its planar structure was unambiguously established by ^1^H-^1^H COSY (Fig. 1 and see Supplementary Fig. S4) correlations of H-6/H-7 and H-5a/H-6a as well as the HMBC (see Supplementary Fig. S5) correlations from CHO-10 to C-4, C-5 and C-6, from H-2 to C-1a, C-2a, C-6a, C-8 and C-9, from CH_3_O-11 to C-2a. Therefore, the structure of compound 1 was established as shown in Fig. 1 and named 2-(3, 4-dihydroxy-2-methoxyphenyl)-1, 3-benzodioxole-5-carbaldehyde (**1**).

**Table 1:** 1H (500 MHz) and ^13^C (125 MHz) NMR spectral data of compound **1** and Reference (*δ*, ppm and *J*, Hz, CD_3_OD).

	Compound 1		Reference	
	^1^H NMR	^13^C NMR	^1^H NMR	^13^C NMR
2	5.18(s)	104.8	5.21(s)	105.3
4	7.28(d, *J* = 1.6 Hz)	115.8	7.31(d, *J* = 2.0 Hz)	115.9
5		131.1		131.3
6	7.27(d, *J* = 1.6 Hz, 6.6 Hz)	126.4	7.31(dd, *J* = 2.0 Hz, 6.5 Hz)	126.9
7	6.89(d, *J* = 8.6 Hz)	116.2	6.91(d, *J* = 7.0 Hz)	116.7
8		147.1		147.6
9		153.7		154.1
10	9.65(s)	193.1	9.68(s)	193.6
11	3.32(s)	49.8		
1a		146.6		147.1
2a		115.3	6.86(d, *J* = 1.5 Hz)	115.3
3a		146.1		146.5
4a		130.8		131.6
5a	6.82(d, *J* = 8.2 Hz)	114.8	6.75(d, *J* = 6.5 Hz)	116.3
6a	6.71(d, *J* = 9.8 Hz)	119.4	6.73(dd, *J* = 1.5 Hz, 7.0 Hz)	119.9

Reference Tagashira *et al*.^35^ reported.

**Figure 2:**
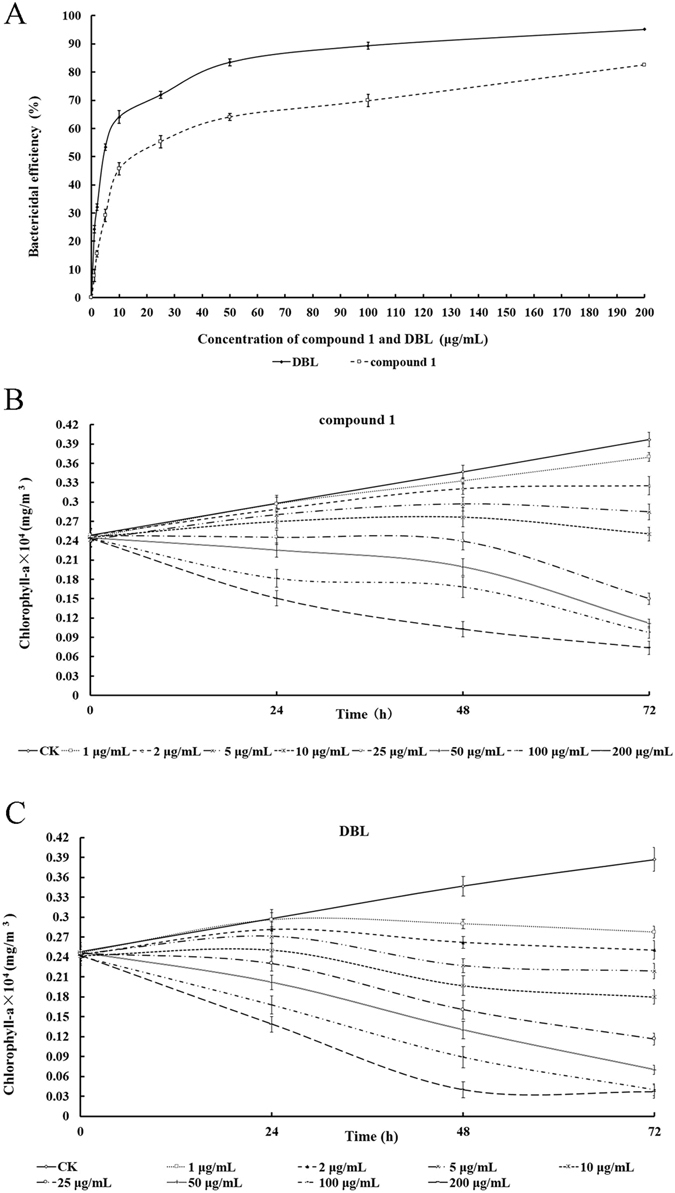
Anticyanobacteria activities of compound **1** and DBL against *M. aeruginosa*. (**A**) The bactericidal efficiency of compound **1** and DBL with different concentrations (1 to 200 μg/mL) against *M. aeruginosa* cells were calculated by measuring the cell density for 72 h; (**B**) The chlorophyll a concentrations of *M. aeruginosa* exposed to compound **1** with different concentrates at 24, 48, 72 h; (**C**) The chlorophyll a concentrations of *M. aeruginosa* exposed to DBL with different concentrates at 24, 48, 72 h. Experiments were performed in triplicate. *Error bars* represent the SD.

Compound **2** was elucidated as 3, 4-dihydroxybenzalacetone (DBL) (Fig. 2, see Supplementary Figs S6–10)^22^: yellow crystal (MeOH) ESI-MS *m/z* 179.2 [M + H]^+^. ^1^H NMR (500 MHz, CD_3_OD) (see Supplementary Fig. S8): δ 7.50 (1 H, d, *J* = 16.1 Hz, H-7), 7.10 (1 H, s, H-5), 6.97 (1 H, d, *J* = 8.2 Hz, H-8), 6.77 (1 H, d, *J* = 8.1 Hz, H-2), 6.53 (1 H, d, *J* = 16.2 Hz, H-6), 2.31 (3 H, s, CH_3_-1); ^13^C NMR (125 MHz, CD_3_OD) (see Supplementary Fig. S9): δ127.7 (C-1), 116.6 (C-2), 146.9 (C-3), 149.9 (C-4), 115.3 (C-5), 124.7 (C-6), 146.9 (C-7), 123.5 (C-8), 201.6 (C-9), 27.0 (C-10).

### Bactericidal activities of compound 1 and DBL

The experimental aim was to determine the inhibitory potency against the growth of *M*. *aeruginosa* by measuring the cell density after exposed to compound **1** and DBL for 72 h, (Fig. 2A). The two compounds exhibited bactericidal activities against an *M. aeruginosa* culture as the cell densities significantly decreased in comparison to that of the control. As the data shown in Fig. 2, DBL has more efficient anti-cyanobacterial activity against *M. aeruginosa*. The EC_50,72h_ values of compound **1** and DBL were 20.6 and 5.1 μg/mL, respectively. The algicidal assay indicated that the anti-*M. aeruginosa* activities of two compounds increased with the dosage.

As shown in the Fig. 2, with the increase of concentration of DBL, the content of chlorophyll a was decreased gradually from 0.28 to 0.04 μg/mL at 72 h, which was 90.45% lower than that of the control. Compound **1** has little inhibitory effect on algae at low concentration, which was 0.37 μg/mL at 1 μg/mL, while the content of chlorophyll a was decreased to 0.07 μg/mL with the increasing concentration of compound **1** (200 μg/mL). According to the OD value, the EC_50_ values of DBL and compound **1** were 5.86 and 18.24 μg/mL, respectively, which were close to the cell density test results. Based on the above, we conclude that compound 1 and DBL can inhibit the growth of *M. aeruginosa* in a dose-dependant manner.

### O_2_^•^^−^ and electric conductivity assay

**Figure 3:**
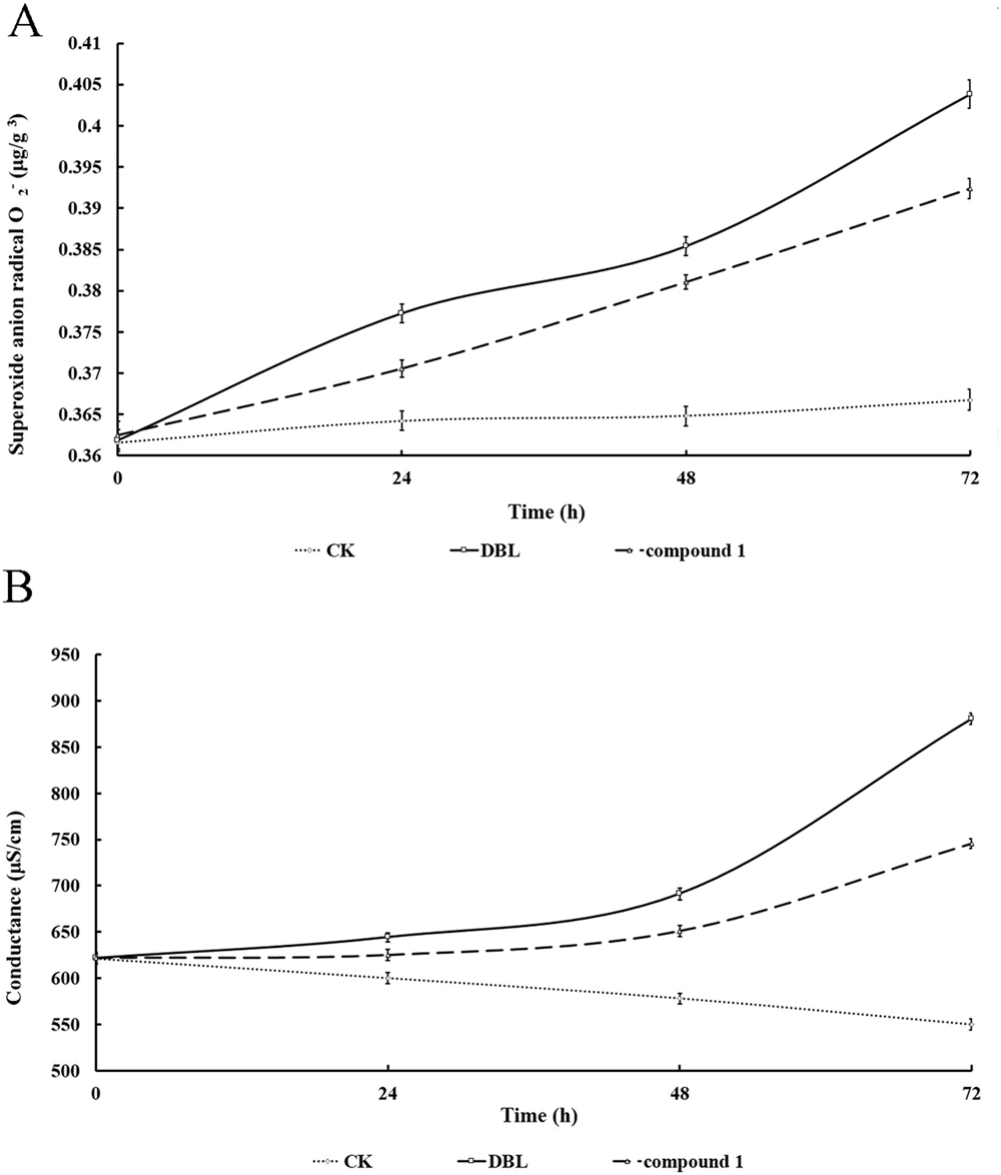
Influences of DBL and compound 1 (4 μg/mL) on O_2_^•−^ and EC of *M. aeruginosa* cells, respectively. All error bars indicate SD of the three replicates. (**A**) The two compounds effect on O_2_^•−^ of *M. aeruginosa* cells, (**B**) The two compounds effect on EC of *M. aeruginosa* cells.

O_2_^•−^ in *M. aeruginosa* cells was induced largely by DBL (4 μg/mL) and the content of O_2_^•−^ increased from 0. 36 ± 0.001 μg/g^3^, which was higher than that of compound **1** with peak ratio of 0.36 ± 0.002 μg/g^3^. The content of O_2_^•−^ in cells exposed to DBL and compound **1** were maximum value of 0.40 ± 0.001 and 0.39 ± 0.001 μg/g^3^ at 72 h (Fig. 3A).

Figure 3B shows effects of two compounds on the electric conductivity (EC) ratio. The EC ratio of DBL was 622 μS/cm initially and increased to 880 ± 6.03 μS/cm on 72 h, which was higher than that of compound **1** (745 ± 5.25 μS/cm). Compared with the control, DBL and compound **1** significantly effected on the EC ratios of *M. aeruginosa*.

### Micro and ultrastructure changes of *M. aeruginosa* exposed to compound 1 and DBL

**Figure 4:**
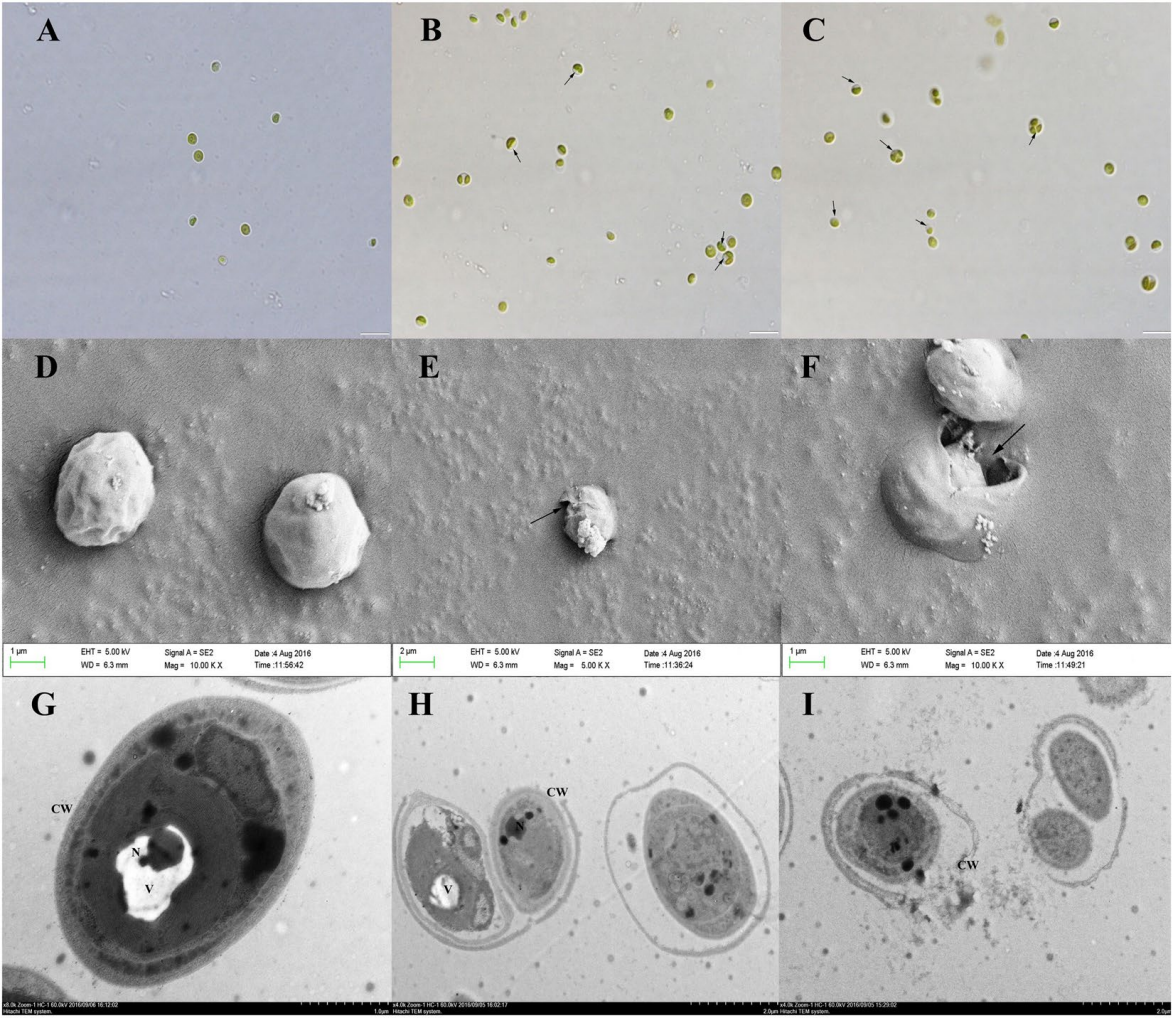
Microstructural and ultramicroscopic structure changes of *M. aeruginosa* exposure to 4 μg/mL compound **1** and DBL for 72 h. Light micrographs of Microcystis cells: (**A**) Normal *M. aeruginosa* cells, scale bar = 20 μm; (**B**) *M. aeruginosa* cells exposed to compound **1**, scale bar = 20 μm; (**C**) *M. aeruginosa* cells exposed to DBL, the arrows in B and C pointed to the shrinkage and perforation of cell membrane, scale bar = 20 μm; SEM of Microcystis cells: (**D**) Control *M. aeruginosa* cells present normal shape, scale bar = 1 μm; (**E**) *M. aeruginosa* cells exposed to compound **1**, scale bar = 1 μm; (**F**) *M. aeruginosa* cells exposed to DBL, scale bar = 0.5 μm; TEM of Microcystis cells: (**G**) Control *M. aeruginosa* cells present normal shape and structure, CW: cell wall, V: vesicles, N: nucleus, scale bar = 1.0 μm; (**H**) *M. aeruginosa* cells exposed to compound **1**, scale bar = 2.0 μm (**I**) *M. aeruginosa* cells exposed to DBL, scale bar = 2.0 μm. Experiments were performed in triplicate.

Our results demonstrated that compound **1** and DBL significantly affected the morphology of *M. aeruginosa* cells. Compared to the control cells (Fig. 4A), the morphological changes of the cells after exposure to 4 μg/mL of compound **1** and DBL were observed under SEM and TEM to evaluate the bactericidal mechanism of tested compounds on morphological micro and ultrastructures (Fig. 4B and C). The *M. aeruginosa* cells appeared to be normal shaped as plump, and round with smooth exteriors in the control (Fig. 4D). After exposure to compound **1** or DBL, majority of *M. aeruginosa* cells exhibited obvious changed in morphology and lost their integrity. Figure 4E and F show that the cytoplasm became notably condensed and plasmolysis occurred in the cells. The untreated cell had complete cell wall and a basic structure, including a nuclear area, vesicle, and other cell organelles (Fig. 4G), whereas the exposed cells were disrupted and lysed. The compounds severely damaged the cell-walls and caused cell disruption, collapsed, perforation and content lysis (Fig. 4H and I). DBL damage was more severe as loss of nuclear area and gas vesicle and is integration of cell architecture.

### Effects on transcription level of *M. aeruginosa* genes

**Figure 5:**
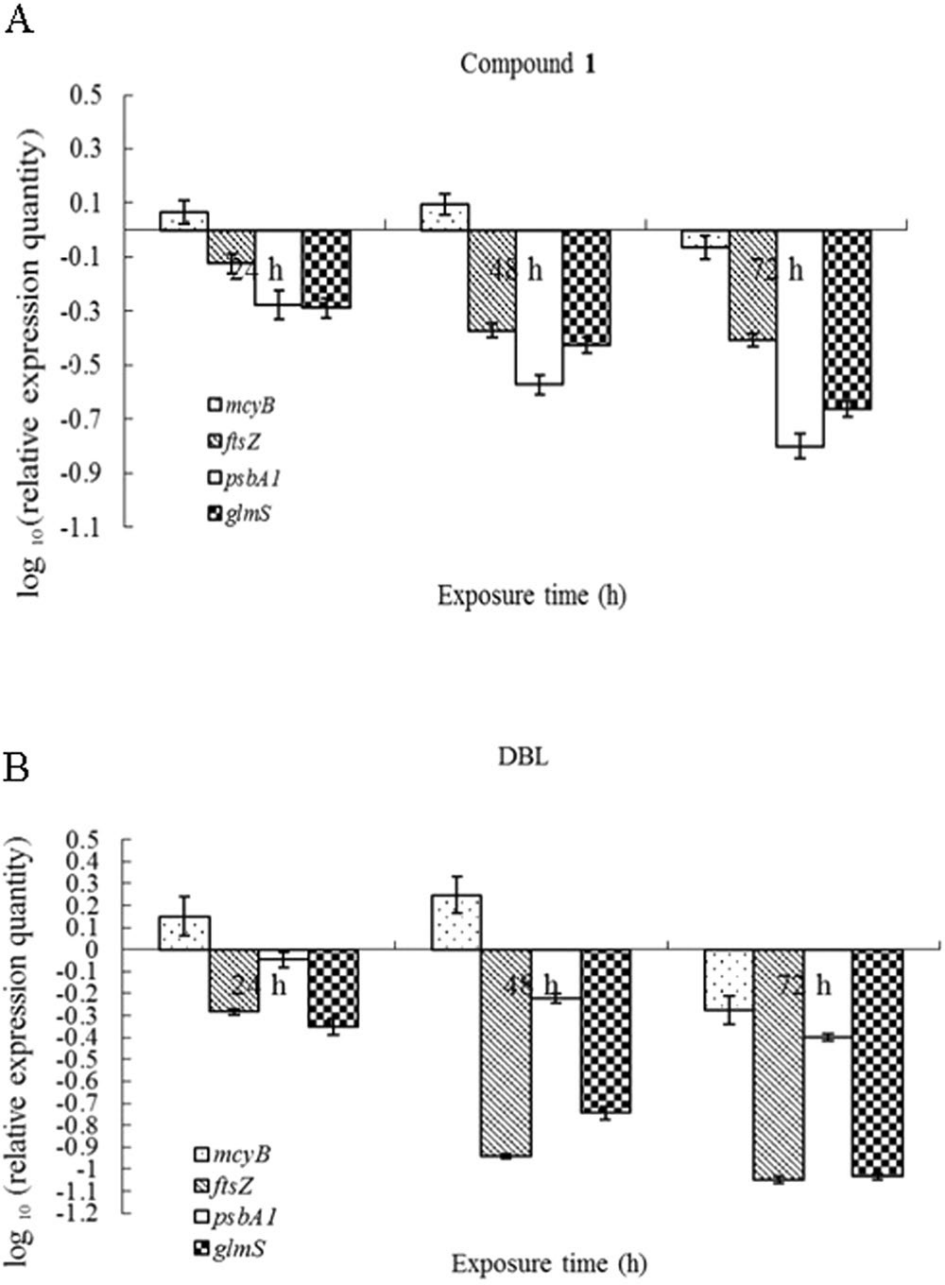
qRT-PCR analysis of the expression of *mcyB*, *ftsZ*, *psbA1* and *glmS* in *M. aeruginosa* exposed to compound **1** (**A**) and DBL (**B**) (4 μg/mL) for 24, 48, and 72 h. The values were normalized to the levels of *16* *S rRNA*, which is a housekeeping gene and an internal reference gene. The y axis values represent the mean expression ± the standard deviations (n = 3) relative to the control. Experiments were performed in triplicate.

Based on the experiments of microscopic observation and determinations of chlorophyll a, electrical conductivity and superoxide anion O_2_^•−^, to further clarify the bactericidal mechanism on gene expression, we tested the key synthesis gene of chlorophyll a and related genes of cell membrane. The four targeted genes, including microcystin in several cyanobacterial generasynthesis genes *mcyB* ^23^, cell division gene *ftsZ*, photosynthesis gene *psbA1*, and peptidoglycan synthesis gene *glmS*, were chosen to analyze the effects of the compound **1** and DBL on gene transcription. We detected the transcriptional expression changes of these genes of *M. aeruginosa* exposed to the two compounds (Fig. 5). Compared to the control, *ftsZ*, *glmS* and *psbA1* genes were slightly down-regulated after 24 h, while expression was reduced significantly after 48 h exposures to compound **1**. The *mcyB* was up-regulated and then reduced. The qRT-PCR analysis demonstrated that DBL increased the transcriptional expressions of *mcyB* then decrease it. Consequently, a decrease in *ftsZ* gene, *psbA1* gene, and *glmS* gene, were observed. The results suggested that DBL seriously influenced the transcription of genes in *M. aeruginosa*.

## Discussion

Previous studies have demonstrated that some microorganisms have powerful algicidal activity against harmful algal blooms^11^. However, only a few algicidal compounds were purified and identified, which included biosurfactants^24^, bacillamides^25, 26^, peptides^27^, proteins^28^, 1-methyl-β-carboline^14^, β-carbolines^7, 29^, and pigments^30^. These algaecides are natural chemicals and, therefore, may be environmentally friendly in controlling HABs^31^.

In this study, we isolated two compounds from *P. noxius* HN-1. Based on the ^1^H and ^13^C NMR spectra the structure of compound **1** is similar to that of the known compound 2-(3′,4′-dihydroxyphenyl)-1,3-benzodioxole-5-aldehyde isolated from *Melissa officinalis* ^32^, differing in a methoxy group is replaced by H at C-11 (δ_C_ 49.8). The known compound is 10-fold more active than ascorbic acid and is easily degraded into two molecules of protocatechualdehyde^32^. Accordingly a hypothesis is suggested that they probably have homogeneous activities.

DBL is a polyphenol derived from the medicinal fungus Chaga (*Inonotus obliquus*) in Japan, and is used as a folk medicine to treat cancers in Russia^33, 34^. DBL has growth-inhibitory effects^35^ and shows strong antioxidant activity in terms of both superoxide and hydroxyl radical scavenging activities^34^, suggesting the therapeutic effects of DBL. However, to our best knowledge, there is no report available on the bactericidal activity of DBL as a natural metabolite produced by *P. noxius*.

The present study is for the first time to show that compound **1** and DBL exhibit anti-cyanobacterial activities against *M. aeruginosa* with EC_50_ values of 20.6 and 5.1 μg/mL. The differential effects of the two compounds may be due to their structural differences. It is similar to other previously reported compounds. It was shown that the EC_50_ values of salcolin A and B isolated from *Hordeum vulgare*, were 6.0 and 9.6 μg/mL against *M. aeruginosa* ^36^. The antialgal allelochemical ethyl 2-methylacetoacetate was isolated from *Phragmites communis* and with the EC_50_ value of 0.65 μg/mL against *M. aeruginosa* ^18^. In addition, compound **1** weakened light result in inhibiting photosynthesis of *M. Aeruginosa*, and the EC_50_ value was close to the cell density test results.

The cell membrane is the target for many antimicrobial agents^37, 38^ and some electrolytes tend to leach out first, then large molecules such as DNA, RNA, and other materials leak out^39^. The release of intracellular components is a good indicator of membrane integrity^38, 39^. In recent studies, it was suggested that some compounds, which act as an environmental stress, can increase the production of O_2_^•−^ in cells^40, 41^. O_2_^•−^ is the precursor of active free radicals that have the potential for reacting with biological macromolecules inducing cell damage.

Exposure to compound **1** and DBL lead to increase of O_2_^•−^ contents in *M. aeruginosa* cells, which may induce lipid peroxidation, indicated the leakage and release of electrolytes, nucleic acids, and proteins from the cyanobacteria and contribute to the increase of EC. Compound **1** and DBL belong to phenolic compounds which are similar to phenolic acid compounds, therefore we infer that target of these two compounds might be the cell membrane. Other report indicated that antioxidant enzyme (superoxide dismutase) activities and specific activities of *A. flos-aquae* were enhanced at the beginning of ρ-hydroxybenzoic acid and ferulic acid oxidative stress conditions^42^.

Although some substances have been reported to control *M. aeruginosa*, their inhibition mechanism remain unknown. Previous studies suggested that those compounds destroy cell structure, cause oxidative damage, and affect algal photosynthesis and enzymatic activities^6, 17, 43^. Zhang *et al*.^44^ demonstrated that 2′-deoxyadenosine produced by *Streptomyces jiujiangensis* strain JXJ 0074^T^ led to severe crumpling, collapse, and perforation of *M. aeruginosa*, and a reduction in chlorophyll content. Bacilysin, isolated from *B. amyloliquefaciens* FZB42, acts against cell walls and also has significant anti-cyanobacterial effects^5^. In the present study, the morphometric analysis at the microstructural and ultrastructural levels by SEM and TEM indicate that compound **1** and DBL primarily affected the cell wall and increase cell permeability, leading to the efflux of intracellular components and eventually cell lysis. Based on the O_2_^•−^ and EC contents assay, *M. aeruginosa* cell membrane was irreversibly damaged under the conditions of two compounds deoxidize stress.

To define the molecular bactericidal mechanism, the expression of microcystin peptide synthesis gene *mcyB*, cell division gene *ftsZ*, photosynthesis gene *psbA1*, and peptidoglycan synthesis gene *glmS* were analyzed by qRT-PCR. The expression abundance of these genes was reduced by compound **1** and DBL and the growth of *M. aeruginosa* was significantly suppressed. Our results are similar to the previous studies that also suggest that the transcript abundance of regulated genes were obviously reduced when *M. aeruginosa* under pyrogallol stress or algicidal bacterium stress^45, 46^. The *ftsZ* gene encodes cell division protein FtsZ, which is essential to the cyanobacterium *Synechocystis* sp. PCC 6803 survival^47^. Combined with cell wall breakage, the decrease in the expression of genes *ftsZ* and *glmS*, indicates that membrane damage may be the bactericidal mechanism for DBL in *M. aeruginosa* cells. Compound **1** had less effect on the cell membrane than DBL.

*M. aeruginosa*, a toxic cyanobacterium, can produce microcystins. Microcystin formation is catalyzed by a complex multifunctional enzyme containing peptide synthetase (*mcyABC*) and hybrid polyketide-peptide synthetase (*mcyDE*)^48^. After *M. aeruginosa* cells were stimulated by compound **1** and DBL, the *mcyB* expression increased, which might be related to increase of the microcystin content caused by release of microcystin from dead *M. aeruginosa* cells. Dziga *et al*. concluded that the expression of *mcyB* is up-regulated under exposure to pyrogallol because of the release of hepatotoxin from dead *Microcystis* cells^49^, which increase microcystin content. Zhang *et al*. have also proved that the transcription expression of the microcystin synthetase gene is affected by ginkgolic acid^6^.

The photosynthetic gene expression is possibly regulated at the transcriptional level^50, 51^. Some studies have indicated that the interruption of the electron transfer chain which affects photosynthetic processes, and oxidant damage may be the inhibitory mechanisms^6, 45, 49, 52^. It has been known that PS II was sensitive to the environment^53^. The reduced abundances of *psbA1* in PS II implies that the repair rate does not keep up with the damage rate and that compound **1** and DBL stress would interfere with electron transport. The *psbA1* gene, the possible target for compound **1**, was significant and rapid downregulated than that of DBL. It may be another factor in the effect on *M. aeruginosa* growth. This result is similar to other compound such as amoxicillin and levofloxacin hydrochloride that decrease PS II activity in *Synechocystis* sp^54, 55^. Based on the qRT-PCR analysis, we suggest that the *psbA1* gene is the potential binding site of compound **1** affecting algal photosynthesis. DBL multisite action, including releasing of microcystin, the cells membrane and cell structure damage, and reduction photosynthesis cause *M*. *aeruginosa* death. The morphological and molecular analysis results indicated that compound **1** and DBL might have different mechanisms against *M*. *aeruginosa* and we will study the protein expression changes in the future to clarify the bactericidal mechanism. In conclusion, compound **1** and DBL, isolated from *P*. *noxius* HN-1, show potent bactericidal activity and may be useful to mitigate harmful algal blooms in a synergistic manner.

## Material and Methods

### General experimental procedures

Thin-layer chromatography (TLC) was performed on silica gel GF254 (Qingdao Haiyang Chemical Co., Ltd, China) and column chromatography was performed with silica gel (60–80, 200–300 mesh, Qingdao Haiyang Chemical Co.). Sephadex LH-20 (Merck, Germany). The ESI-MS spectra were measured with a VG Auto-3000 Spectrometer, Sephadex LH-20 (Merck, Germany) and MS-C18 column (3.5 μm, 4.6 by 150 mm, Waters). Nuclear Magnetic Resonance (NMR) spectra were obtained on a Bruker AV-500 spectrometer with tetramethylsilane (TMS) as an internal standard. Infrared Spectroscopy (IR) spectra were recorded on a Nicolet 380 FT-IR instrument, as KBr pellets (Thermo, Pittsburgh, PA, USA). UV spectra were obtained on a Shimadzu UV-2550 spectrometer (Beckman, Brea, CA, USA).

### Microorganisms

*P. noxius* strain HN-1 was isolated from brown root pathogens collected in Changjiang city, Hainan Province, China^21^, was cultured in potato dextrose agar (PDA) medium at 28 °C and stored in our lab (see Supplementary Fig. S1). The strain HN-1 was deposited in China Center for Type Culture Collection (CCTCC) (CCTCC M 2016242) (GenBank accession number KX592167).

### Isolation and identification of the compounds

*P. noxius* strain HN-1 was cultured on PDA at 28 °C for 7 days. Two pieces of mycelial agar plugs (0.5 cm × 0.5 cm) were inoculated into 1 L Erlenmeyer flasks containing 400 mL potato dextrose broth (PDB). The cultivation was shaken at 120 r/min at 28 °C for 7 days, and then kept in still at 28 °C for 45 days. The culture broth (60 L) was filtered to give the filtrate and mycelia. The crude extract was reduced *in vacuo* to approximately 1 L and partitioned in succession between H_2_O and petroleum ether, ethyl acetate (EtOAc) and n-butyl alcohol^56^. The EtOAc extract (1.26 g) was separated on a silica gel column (200–300 mesh) with sequential gradient elution with 100% chloroform (CHCl_3_), a mixture of CHCl_3_/methanol (MeOH) (100:1, 50:1, 25:1, 15:1, 10:1, 5:1, 2:1, 1:1, *v/v*), and finally 100% MeOH into 10 fractions. The resulting fractions were combined according to TLC profiles on silica gel GF254 (Marine Chemical Industry Factory, Qingdao, China). Based on the bioassay, the fractions were tested for inhibition against *M. aeruginosa*. Fraction 5 (Fr.5) (180 mg) was submitted to chromatography and further separated *via* Sephadex LH-20 and on silica gel column with CHCl_3_/MeOH (10:1, *v/v*), yielding the compound **1** (21.57 mg). Fraction 4 (Fr.4) (110 mg) was submitted to chromatography on a silica gel column with CHCl_3_/MeOH (15:1, *v/v*) as an eluent and further separated by chromatography *via* Sephadex LH-20 column with ethanol as an eluent and on a silica gel column with CHCl_3_/MeOH (20:1, *v/v*), yielding DBL (15.78 mg).

### *M. aeruginosa* culture

*M. aeruginosa* NIES-843 was purchased from the Freshwater Algae Culture Collection of the Institute of Hydrobiology (Wuhan, China) and cultured in sterilized BG11 medium at 25 ± 1 °C under a 12 h: 12 h (light: dark) cycle with 60 μmol photons m^−2^ s^−1^ ^5^.

### Bioassay

Compound **1** or DBL was added to the cultures of *M. aeruginosa* (1 × 10^7^ cells/mL) with the final concentrations of 1, 2, 5, 10, 25, 50, 100, 200 μg/mL, and cultivated at 25 °C under 40 μmol photons/(m^2^ s) and a 12 h:12 h (light: dark) cycle. The bactericidal activities of compound **1** and DBL against *M. aeruginosa* were assayed according to the procedure described by Li *et al*.^57^. A control was tested using sterile water inoculation. The number of cells was observed under Olympus BX51 (Olympus, Japan).

*M. aeruginosa* samples (20 mL) were centrifuged at 3,500 × g for 20 min and then extracted in 90% acetone for 24 h at 4 °C, and the supernatant removed into a 10 mL volumetric flask and diluted with 90% acetone to 10 mL, after which the chlorophyll a concentrations were determined by using the following equation: chlorophyll a concentration (mg/m^3^) = [11.64 × (OD_663_ − OD_750_) − 2.16 × (OD_645_ − OD_750_) + 0.1 × (OD_645_ − OD_750_)] × 1/2.

The bactericidal activities of compound **1** and DBL were calculated by the following equation: bactericidal efficiencies (%) = (1 − treatment/control) × 100, where the treatment and control are cell densities of *M*. *aeruginosa* with and without compound **1** or DBL inoculation, respectively^58^. *M*. *aeruginosa* growth was monitored at 72 h. The EC_50,72h_ values (i.e., median lethal concentration relative to the control) were obtained from the sigmoidal inhibition curves fitted by probit regression analysis (SPSS 19.0). To verify the reliability of the experimental results, all the experiments were carried out three times, and good repeatability was obtained.

### The measurement of electric conductivity

Compound **1** or DBL was added to the cultures of *M. aeruginosa* (1 × 10^7^ cells/mL) with the final concentration of 4 μg/mL for 72 h. The electric conductivity (EC) was analyzed using a portable conductivity meter (Cole-Parmer Instrument Company, USA). Five milliliters of each sample was taken from the culture flask and was immediately filtered with a 0.22 μm Millipore^59^. The supernatant was used for analysis.

### Measurement Method for Superoxide Anion Radical

Compound **1** or DBL was added to the cultures of *M. aeruginosa* (1 × 10^7^ cells/mL) with the final concentration of 4 μg/mL for 72 h. *M. aeruginosa* sample (20 mL) was centrifuged at 4000 × g for 20 min and then was homogenized with ice-cold phosphate buffered saline (PBS) (6 mL, 65 mM, pH 7.8), filtered with filter paper, and centrifuged at 5000 × g for 10 min at 4 °C. 2 mL supernatant was added to 1.5 mL PBS (65 mM, pH 7.8) and 0.5 mL hydroxylamine hydrochloride (10 mM), followed by incubation at 25 °C for 20 min. After that, 2 mL of the mixture was added to 2 mL sulfanilic acid (17 mM) and 2 mL α-naphthylamine (17 mM), incubated for 20 min at 25 °C. The samples were settled for 10 min at room temperature and was measured at 530 nm. O_2_^•−^ was determined by using the following equation: M (μg/g^3^) = 2 × V_t_ × n/(F_W_ × V_S_), n is concentration of NO_2_^−^ (μg/mL), V_t_ is total volume, F_W_ is weight of sample, V_S_ is the crude enzyme extract volume^59^.

### Cells microstructure and ultrastructural analysis

Scanning electron microscopy (SEM) and transmission electron microscopy (TEM) analysis were used to test the influence of compound **1** and DBL. *M. aeruginosa* was cultivated at 25 °C under 60 μmol photons/(m^2^ s) and a 12 h:12 h (light: dark) cycle. The cells were exposed to compound **1** and DBL with the final concentration of 4 μg/mL for 72 h, respectively. The fixed cells were collected by centrifugation, prefixed in 2.5% glutaraldehyde and washed three times in 0.1 M phosphate buffer for 10 min. Dehydration was done with a gradient series of ethanol. For SEM analysis samples, the cells were coated with gold, and examined with a Hitachi S-3000N SEM (Hitachi, Japan). For TEM analysis samples, the cells were postfixed in 1% osmium tetroxide for 1 h and dehydrated with a gradient series of ethanol. After dehydration, the samples were embedded in Epon 812 and sectioned with an ultramicrotome (LKB-V, Sweden). The sections were examined under a Hitachi H-600 TEM (Hitachi, Japan). Micrographs were taken at 10.0 kV^5^.

### qRT-PCR Analysis

*M. aeruginosa* was exposed to 4 μg/mL compound **1**, DBL or water as the control for 24 , 48 , 72 h. After incubation, the cells were collected by centrifuging at 10,000 rpm for 10 min at 4 °C. Total RNA was extracted with TRIzol reagent (Invitrogen, USA). cDNA was synthesized with the reverse transcriptase kit (TaKaRa Bio Inc, Dalian, China). qRT-PCR was performed with SYBR Premix Ex Taq (TaKaRa Bio) and an ABI 7500 Fast Real-Time PCR Detection System in a 20 μL volume. The conditions consisted of one cycle of 3 min at 95 °C followed by 40 cycles of 95 °C for 15 s, 56 °C for 30 s. Primers of target genes were listed in Table 2 and the *16* *S rRNA* gene was used as the internal reference for normalization.

**Table 2:** Primers designed for qRT-PCR analysis.

Gene name	Sequence (5′–3′)
*16* *S rRNA*	F: GGACGGGTGAGTAACGCGTA R: CCCATTGCGGAAAATTCCCC
*glmS*	F: TGTGCCTCCGATGTCAGT R: ATGAAGTGACGATAACCCT
*psbA1*	F: GGTCAAGARGAAGAAACCTACAAT R: GTTGAAACCGTTGAGGTTGAA
*mcyB*	F: CCTACCGAGCGCTTGGG R: GAAAATCCCCTAAAGATTCCTGAGT
*ftsZ*	F: TCGCTGCTATTTCCTCGC R: TGACTTCTCCCTGCATTTTCT

Reference Wu *et al*.^5^ reported.

### Statistical analysis

All experiments were at least in triplicate. Statistical analyses were performed with SPSS 19.0. qRT-PCR data were analyzed by the 2^−△△CT^ method. The mean value and standard deviation (SD) of the three replicates were calculated.

## Electronic supplementary material

supplymentary information
