# Supplementary material for: Bactericidal metabolites from Phellinus noxius HN-1 against Microcystis aeruginosa
Source: Sci Rep. 2017 Jun 9;7:3132. doi: 10.1038/s41598-017-03440-2 (PMC5466663; doi:10.1038/s41598-017-03440-2)
Supplement: Supplementary file 1 — supplymentary information [file 41598_2017_3440_MOESM1_ESM.pdf]

Bactericidal matebolites from *Phellinus noxius* HN-1 against *Microcystis aeruginosa*

Pengfei Jin[1#] Haonan Wang[1#] Wenbo Liu[1] Shujian Zhang[2] Chunhua Lin[1] Fucong Zheng[1] Weiguo Miao[1]*

1.College of Environment and Plant Protection, Hainan University, Haikou 570228, China

2.Department of Plant Pathology, University of Florida, Gainesville, FL 32611-0680, United States of America

*****Corresponding author: Prof Weiguo Miao, tel/fax: 86-898-66270229 E-mail: miao@hainu.edu.cn [#]These authors contributed equally to this work

Fig. S1. The morphology of *Phellinus noxius* HN-1. A: The colony of *P. noxius* on potato dextrose agar; B: The trichocysts of *P. noxius* produced on PDA

Fig. S2 ESI-MS spectra of compound **1** (CD3OD)

Fig. S3. ^{1}H NMR spectrum of compound **1**

Fig. S4 ^{13}C NMR spectrum of compound **1**

Fig. S5 ^{1}H-^{1}H COSY spectrum of compound **1**

Fig. S6 HMBC spectrum of compound **1**

Fig. S7 ESI-MS spectra of DBL (CD3OD)

Fig. S8 ^{1}H NMR spectrum of DBL

Fig. S9 ^{13}C NMR spectrum of DBL

Fig. S10 HMBC spectrum of DBL

Fig. S11 ^{1}H-^{1}H COSY spectrum of DBL

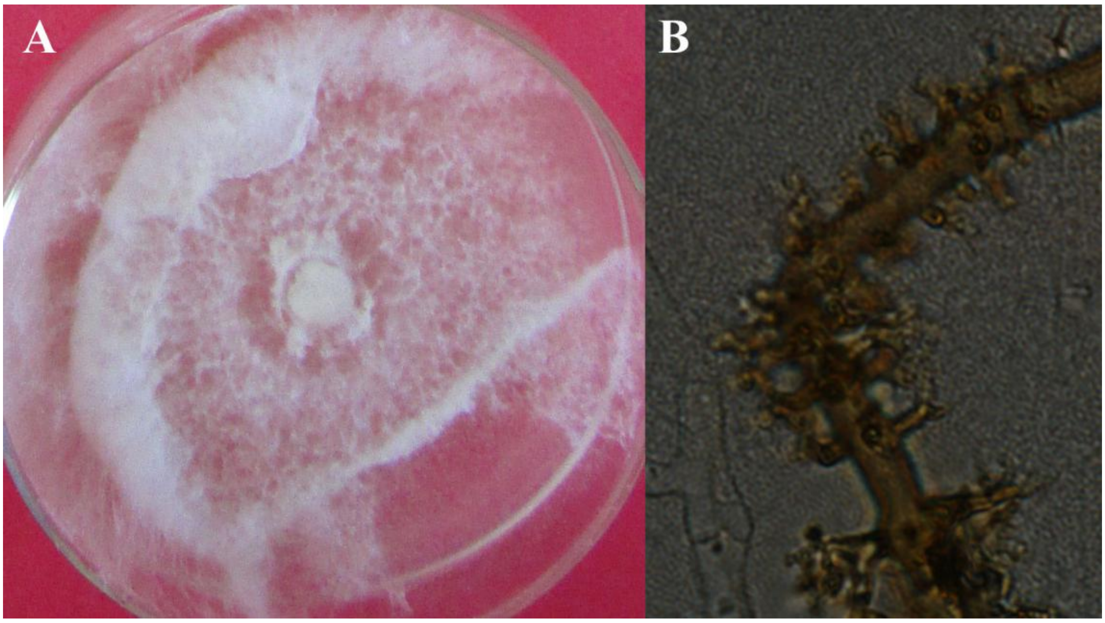

Fig. S1 The morphology of *P. noxius* NH-1. A: The colony of *P. noxius* on potato dextrose agar; B: The trichocysts of *P. noxius* produced on PDA

Qualitative Analysis Report

Data Filename	Compound1.d	Sample Name	Compound1
Sample Type	Sample	Position	P1-C1
Instrument Name	Instrument 1	User Name	
Acq Method	SIBU.m	Acquired Time	6/8/2016 10:09:43 AM
IRM Calibration Status	Success	DA Method	ESI+.m
Comment			

Sample Group		Info.	
Acquisition SW	6200 series TOF/6500 series		
Version	Q-TOF B.05.01(B5125.2)		

User Spectra

Fragmentor Voltage	Collision Energy	Ionization Mode
135	0	ESI

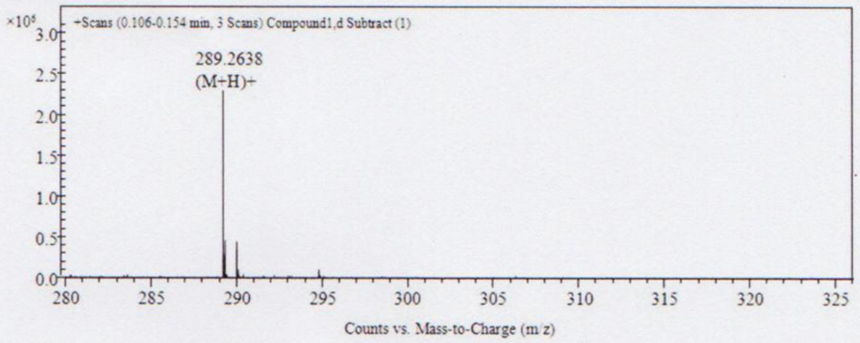

Peak List

m/z	z	Abund	Formula	Ion
289.2638	1	25127435.86	C15 H12 O6	(M+H)+

Formula Calculator Element Limits

Element	Min	Max
C	15	15
H	0	20
O	0	20

Formula Calculator Results

Formula	CalculatedMass	CalculatedMz	Mz	Diff.(mDa)	Diff.(ppm)	DBE
C15 H12 O6	288.2522	289.2624	289.2638	-1.4	-1.4	19.000

---End Of Report---

Fig. S2 ESI-MS spectra of compound **1** (CD_3OD)

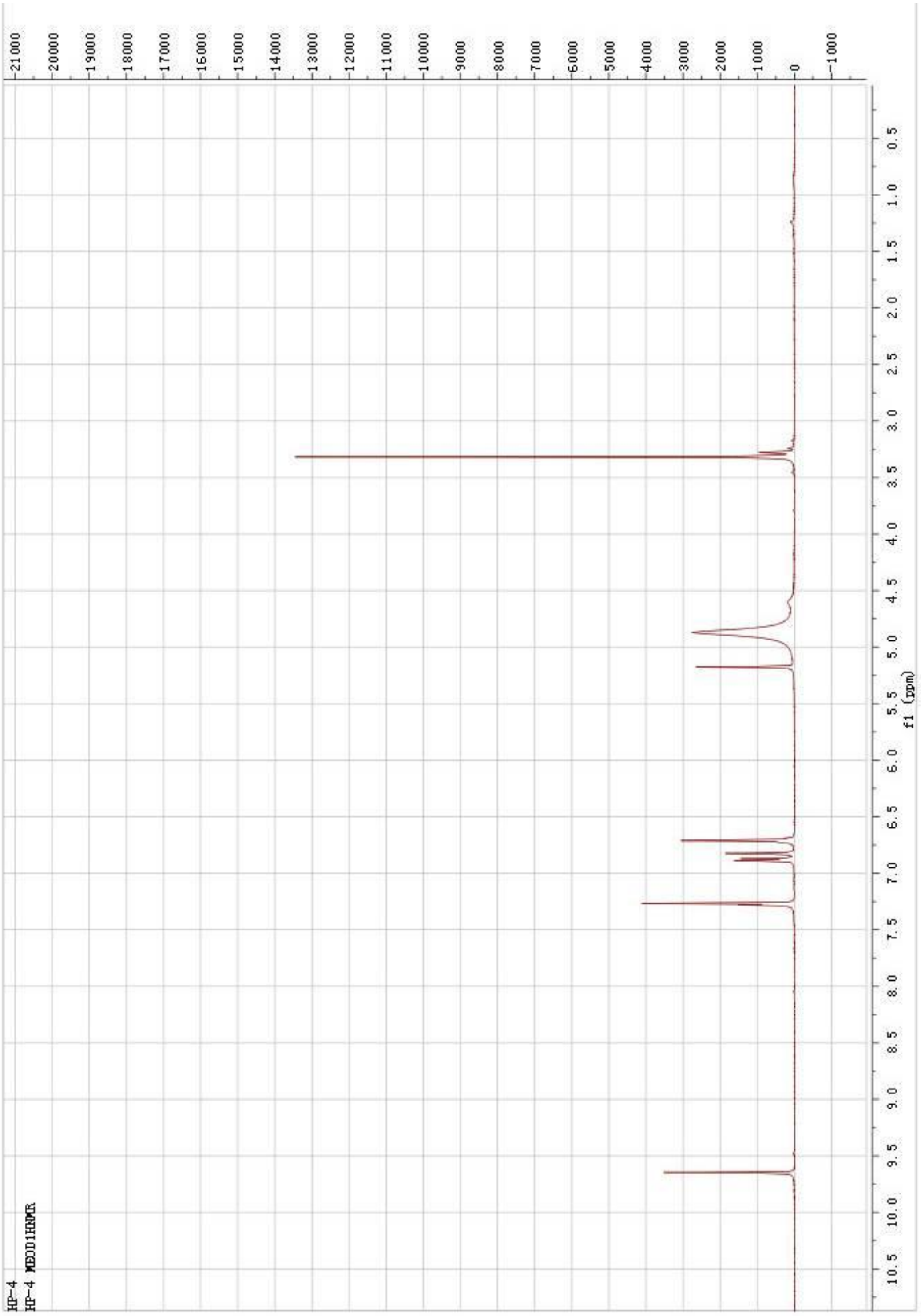

Fig. S3 ^{1}H NMR spectrum of compound **1**

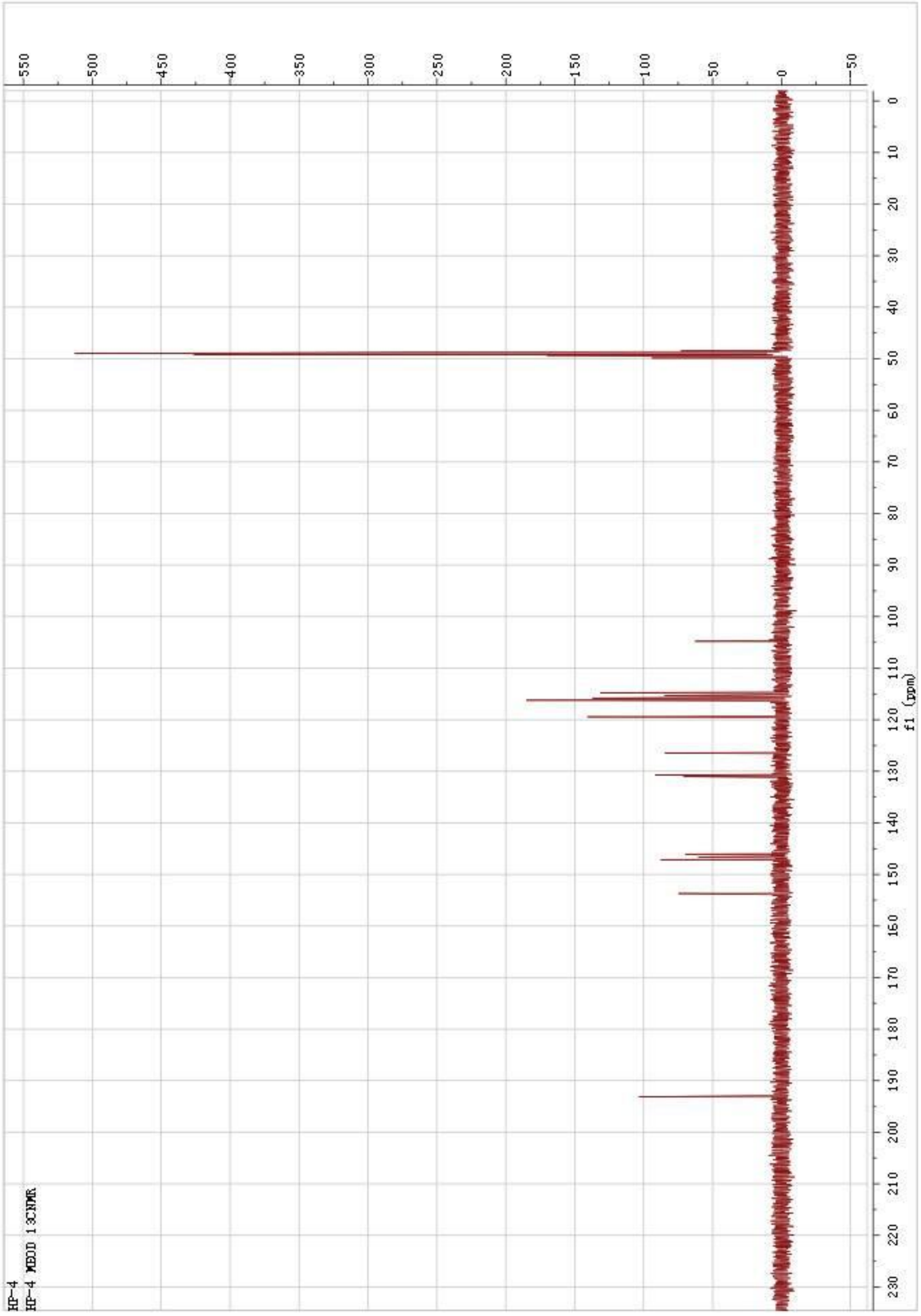

Fig. S4 ^{13}C NMR spectrum of compound **1**

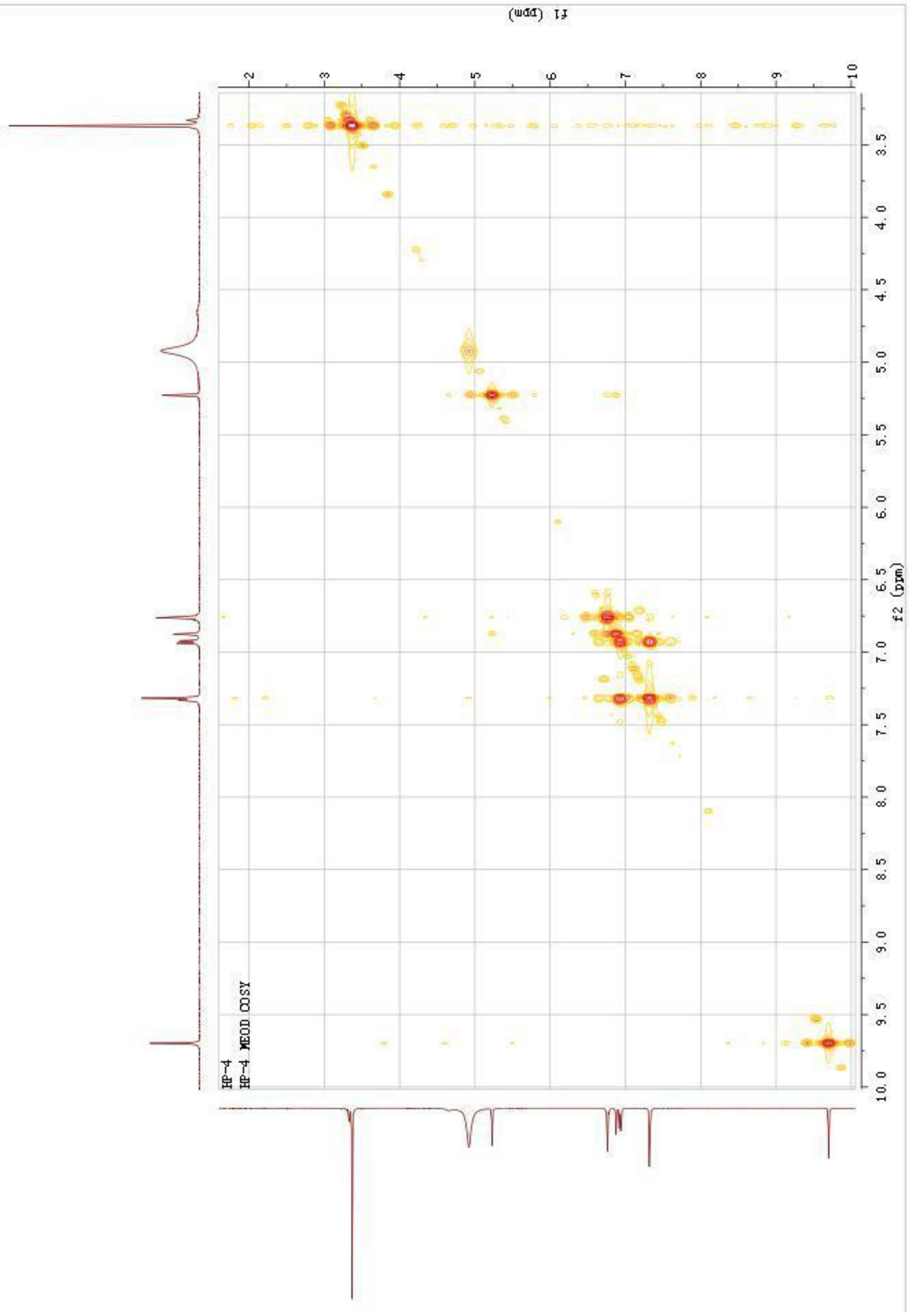

Fig. S5 ^{1}H-^{1}H COSY spectrum of compound **1**

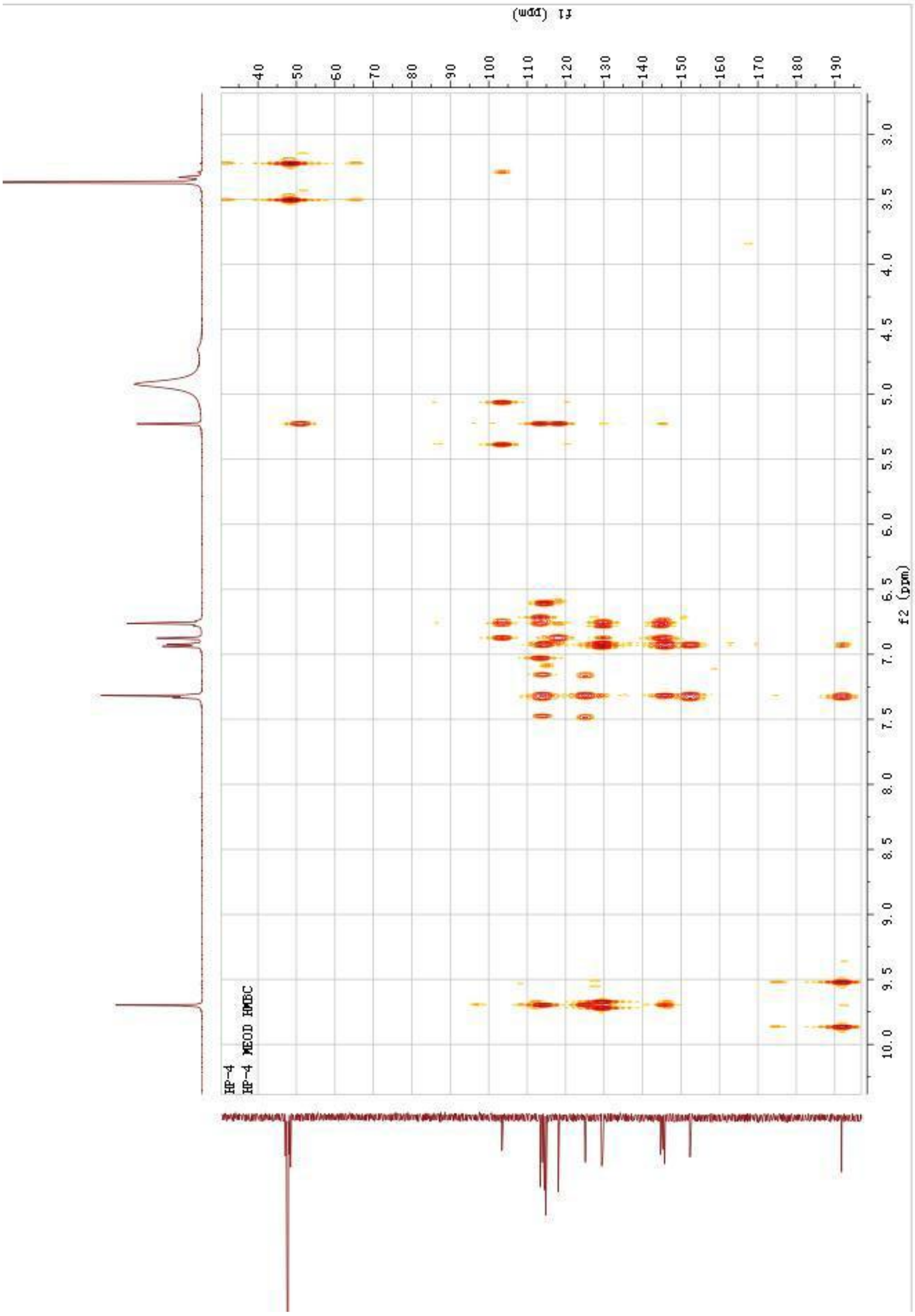

Fig. S6 HMBC spectrum of compound **1**

Mass Spectrum List Report

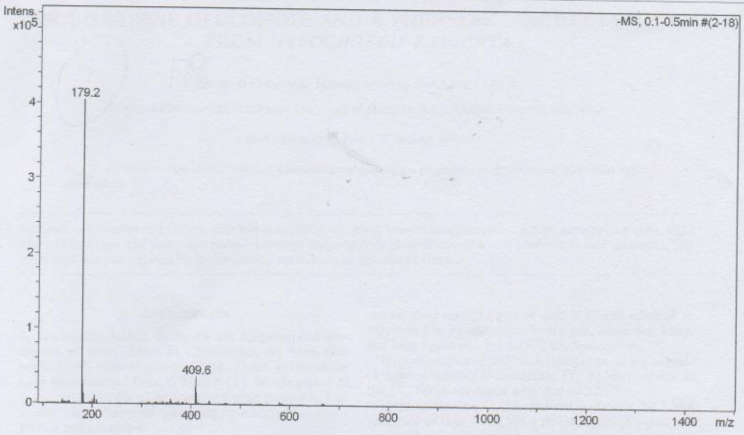

m/z	z	I	FWHM
179.2		403900	
179.9		31792	
180.4		25580	
181.7		13222	
204.6		10305	
205.4		7080	
409.0		23566	
409.6		36722	
410.2		5683	
411.3		15876	

Fig. S7 ESI-MS spectra of DBL (CD_3OD)

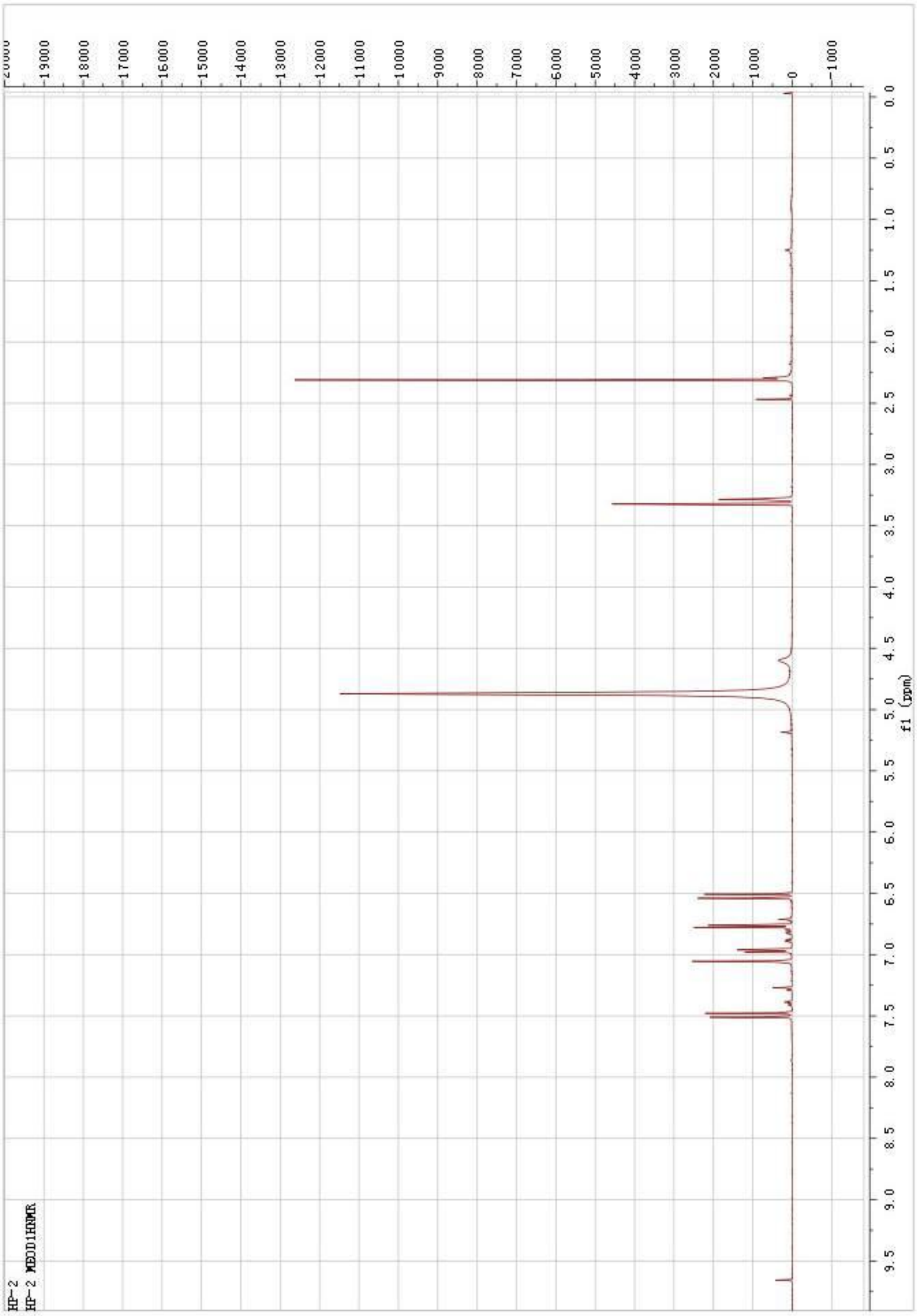

Fig. S8 ^{1}H NMR spectrum of DBL

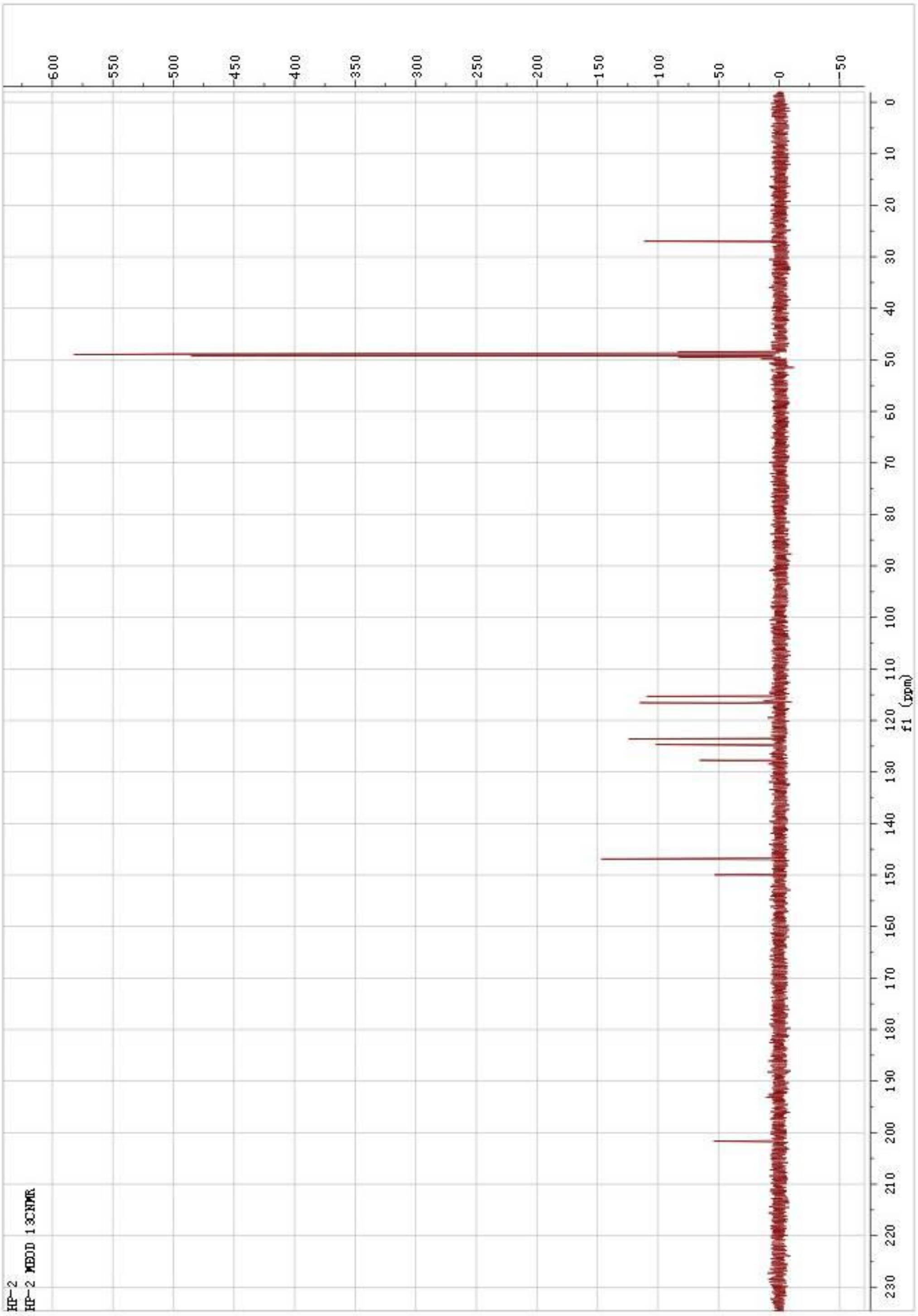

Fig. S9 ^{13}C NMR spectrum of DBL

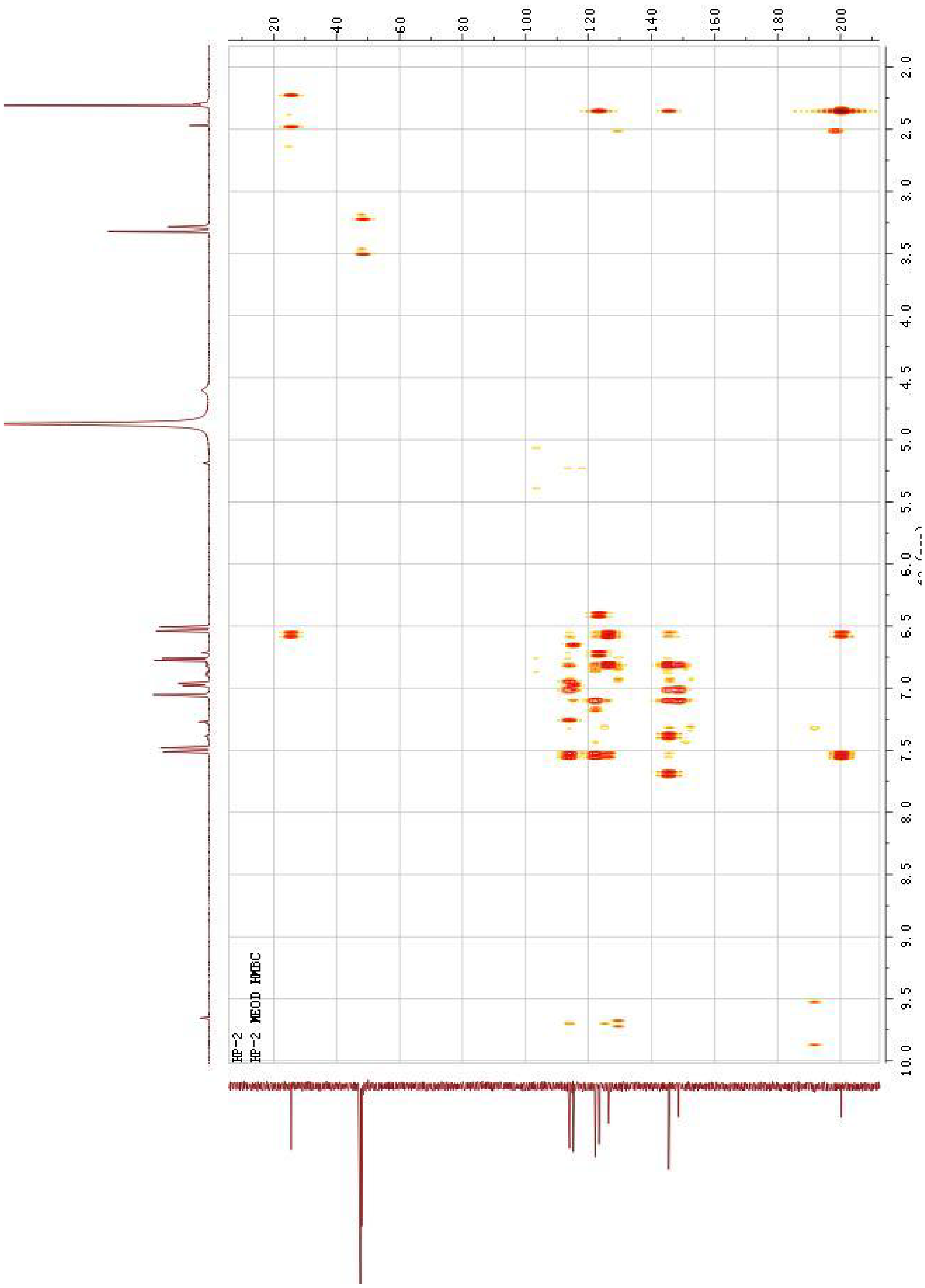

Fig. S10 HMBC spectrum of DBL

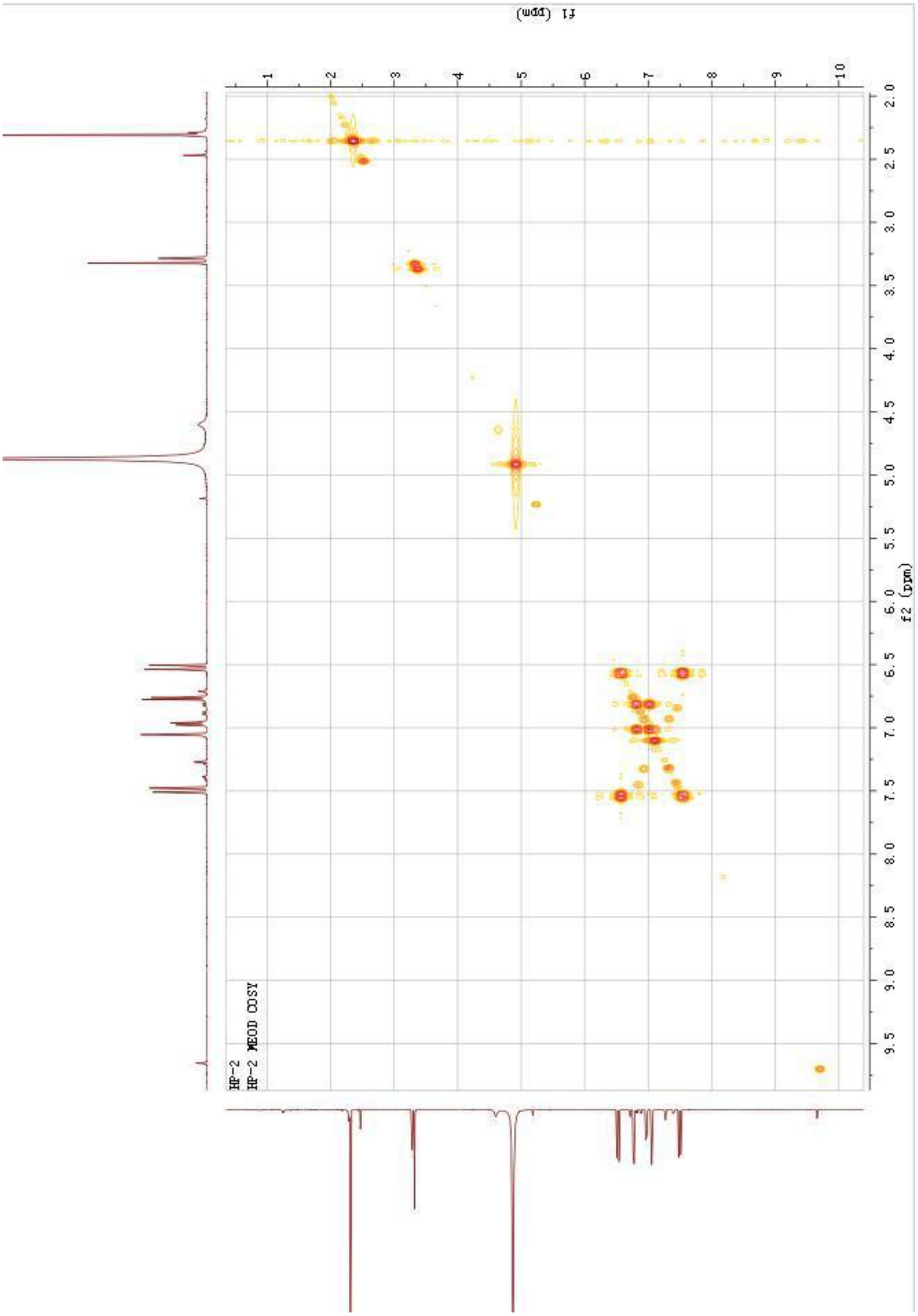

Fig. S11 ^{1}H-^{1}H COSY spectrum of DBL
